# Hyperglycemia exacerbates dengue virus infection by facilitating poly(A)-binding protein–mediated viral translation

**DOI:** 10.1172/jci.insight.142805

**Published:** 2022-11-08

**Authors:** Ting-Jing Shen, Chia-Ling Chen, Tsung-Ting Tsai, Ming-Kai Jhan, Chyi-Huey Bai, Yu-Chun Yen, Ching-Wen Tsai, Po-Chun Tseng, Chia-Yi Yu, Chiou-Feng Lin

**Affiliations:** 1 Graduate Institute of Medical Sciences, Taipei Medical University, Taipei, Taiwan.; 2 Department of Microbiology and Immunology, School of Medicine, Taipei Medical University, Taipei, Taiwan.; 3 School of Respiratory Therapy, College of Medicine, Taipei Medical University, Taipei, Taiwan.; 4 Research Center of Biostatistics, College of Management, Taipei Medical University, Taipei, Taiwan.; 5 Epidemic Intelligence Center, Taiwan Centers for Disease Control, Taipei, Taiwan.; 6 Ministry of Health and Welfare, Institute of Health Policy and Management, College of Public Health, National Taiwan University, Taipei, Taiwan.; 7 National Institute of Infectious Diseases and Vaccinology, National Health Research Institutes, Miaoli, Taiwan.; 8 Center of Infectious Diseases and Signaling Research, National Cheng Kung University, Tainan, Taiwan.

**Keywords:** Infectious disease, Virology, Diabetes, Mouse models, Translation

## Abstract

Diabetes mellitus (DM) is highly comorbid with severe dengue diseases; however, the underlying mechanisms are unclear. Patients with DM have a 1.61-fold increased risk of developing dengue hemorrhagic fever. In search of host factors involved in dengue virus (DENV) infection, we used high-glucose (HG) treatment and showed that HG increased viral protein expression and virion release but had no effects on the early stages of viral infection. After HG stimulation, DENV–firefly luciferase–transfected assay and cellular replicon–based assay indicated increased viral translation, whereas using the glucose uptake inhibitor phloretin blocked this effect. HG treatment increased the translational factor poly(A)-binding protein (PABP) in a glucose transporter–associated, PI3K/AKT-regulated manner. Silencing PABP significantly decreased HG-prompted virion production. HG enhanced the formation of the PABP–eukaryotic translation initiation factor 4G complex, which is regulated by protein–disulfide isomerase. Hyperglycemia increased PABP expression, mortality rate, viral protein expression, and viral loads in streptozotocin-induced DM mice. Overall, hyperglycemic stress facilitates DENV infection by strengthening PABP-mediated viral translation.

## Graphical abstract

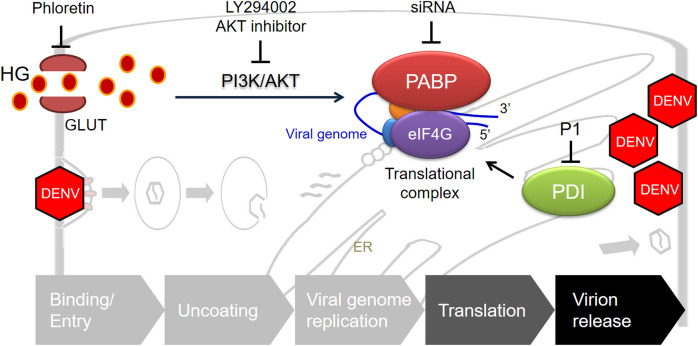

## Introduction

*Aedes* mosquitoes transmit the dengue virus (DENV) and cause outbreaks worldwide, especially in tropical and subtropical regions. They are estimated to have infected 390 million people and to induce millions of fatal cases each year (1, 2). The disease severity has a broad spectrum, ranging from mild febrile illness, called dengue fever (DF), to the more severe dengue diseases, including dengue hemorrhagic fever (DHF), dengue shock syndrome, CNS impairment, and multiple organ involvement (3). Unfortunately, it is difficult to develop vaccines to protect humans from secondary infection because of Ab-dependent enhancement of infection, which may cause severe disease from different DENV serotypes (4, 5). Additionally, there are no effective antiviral drugs to treat patients with dengue, because of the delayed treatment and side effects of chemical agents (6). Therefore, it is urgent to identify and target pathogenic factors to defend against DENV infection.

Underlying diseases, such as diabetes mellitus (DM) and cardiovascular dysfunction, are reported to be comorbid with high prevalence in patients with severe DENV infection (7, 8). Type 1 DM is a metabolic disease in which individuals lose the ability to produce insulin, due to autoimmune destruction of insulin-producing β cells. Type 2 DM is generally due to genetic defects, aging, not enough exercise, and obesity that lead to insulin resistance and the deficiency of insulin production (9). Patients with dengue and with diabetes have an elevated risk of developing severe organ involvement outcomes compared with patients with dengue without diabetes (10–13). For viral infections, a high glucose (HG) level enhances HIV type 1 entry into T cells by increasing CXC chemokine receptor type 4 expression (14). Hyperglycemic stress facilitates West Nile virus infection and induces paralysis-related mortality by impairing immune responses in type 2 diabetic mice (15). Moreover, a higher OR of in-hospital death in patients with DM who were infected with COVID-19 was noticed (16). Hyperglycemia is suggested to exacerbate COVID-19 infection by potentially increasing the concentrations of glycosylated angiotensin-converting enzyme receptor 2 and glycosylated viral spike protein (17). Hyperglycemia, a diabetic condition defined by an increase in blood glucose level, may promote DENV infection and pathogenesis. However, the possible pathogenic mechanisms of HG-exacerbated DENV infection are still unclear.

After binding to and entering host cells, DENV is uncoated to replicate its genome in the cytoplasm, accompanied by the translation of viral proteins in the endoplasmic reticulum. DENV virions are then packaged and released from infected cells (18). Host factors such as RNA-binding proteins are required for DENV replication. Nuclear factor 90, polypyrimidine tract-binding protein (PTB), and heterogeneous ribonucleoprotein (hnRNP) C1/C2 promote DENV replication by assisting RNA synthesis (19, 20). Moreover, the silencing of hnRNP K results in reduced DENV multiplication (21). Human eukaryotic translation elongation factor 1A (eEF1A) and human La autoantigen can interact with the 3′-UTR of DENV to favor viral RNA replication (22).

In contrast to the repressive role of Y box–binding protein 1 (YB-1), P100 and poly(A)-binding protein (PABP) interact with the 3′ stem-loop of DENV to enhance the efficacy of viral translation (23–25). PABP is a host translational factor that interacts with eukaryotic translation initiation factor 4G (eIF4G) and eIF4B to stabilize the translational complex (26). Because of its crucial role in protein translation, PABP also has roles in viral infection. The influenza virus protein NS1 enhances viral translation by interacting with eIF4G, PABP1, and viral mRNA (27). The PABP–eIF4G interaction also stimulates the internal ribosome entry site–dependent translation of rotavirus infection (28). PABP also interacts with protein-disulfide isomerase (PDI), followed by binding to the 5′-UTR of insulin mRNA to increase protein translation and the production of insulin in β cells under HG stimulation (29). In this study, we investigated the possible host factors that promote DENV infection in HG-treated cells and further evaluated potential antiviral strategies by blocking glucose uptake in vitro and in vivo.

## Results

### Patients with DM have an increased risk of developing severe DENV disease.

Data for this study were obtained from the National Health Insurance Research Database (NHIRD) using records from the Taiwan Centers for Disease Control (CDC). We screened data from 31,270 patients with dengue (case group) from 1998 to 2014 and enrolled 30,944 people in this study according to the criteria described in Methods. Of these eligible patients, 3,299 had a confirmed DM diagnosis (DM exposed) 1 year before the DENV infection date, and 27,645 participants did not have DM (DM unexposed). For the control group, we enrolled 123,776 participants who had no dengue disease diagnosis from 2000 to 2014. Among these eligible participants, 12,579 had a confirmed DM diagnosis 1 year before the index date, and 111,197 were not patients with DM. The case group was matched 1:4 to the control group for age, sex, and residence (Supplemental Figure 1; supplemental material available online with this article; https://doi.org/10.1172/jci.insight.142805DS1). For these participants, the mean age was comparable (44.72 years for both groups; *P* = 0.99). There was also no significant difference between the case and control groups for sex (*P* = 1.00). The mean Charlson Comorbidity Index score was 0.68 and 0.63 for the case and control groups, respectively (*P* < 0.001), indicating the high association of comorbidity and DENV infection. Hypertension (19.35%), hyperlipidemia (14%), and DM (10.66%) were the most associated comorbidities in DENV-infected individuals (*P* < 0.001) (Supplemental Table 1).

We further analyzed the prevalence of DENV infection among patients with DM. Of all patients with DM, 3,299 were diagnosed with DENV infection, and 12,579 were classified as control participants (OR: 1.06). Among the DENV-infected patients with DM, 3,182 cases were diagnosed with DF (OR: 1.05) and 117 cases were diagnosed with DHF (OR: 1.61). After adjusting for age, sex, residence, and Charlson Comorbidity Index score, although the multivariable analysis showed a decreased OR (0.96) of diagnosis with the mild DENV disease in patients with DF and DM, there was still a notable 1.44-fold increased risk of DHF diagnosis among all patients with DM (Table 1). These results reveal that DM is comorbidity highly associated with severe DENV disease and that hyperglycemia may promote DENV infection.

### HG enhances DENV infection.

**Figure 1:**
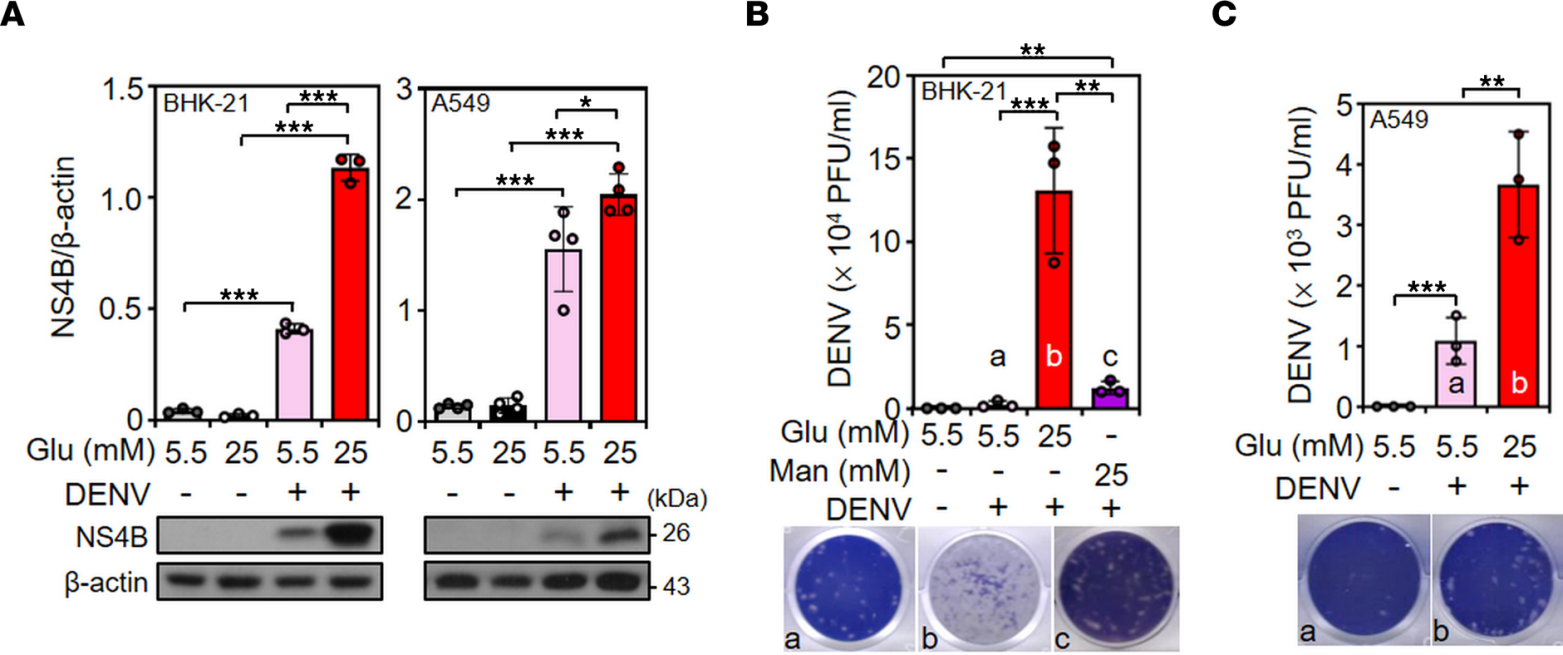
HG treatment increases DENV NS4B expression and virion release. (**A**) Representative Western blot showing the expression of the viral protein NS4B in BHK-21 cells and A549 cells pretreated with 5.5 or 25 mM glucose-containing (Glu-containing) medium for 48 hours and then infected with DENV for 48 hours. Plaque assays were conducted to determine the viral titer in BHK-21 (**B**) and A549 (**C**) cells. Mannose (Man) was used as a control. The mean ± SD quantitative data of at least 3 independent experiments are reported. *P < 0.05, **P < 0.01, ***P < 0.001.

To mimic DENV infection under hyperglycemic stress, baby hamster kidney (BHK) fibroblast BHK-21 cells that had been maintained for 1 month were persistently treated with 5.5 mM glucose in culture medium or alternatively treated with 25 mM glucose for 48 hours (Supplemental Figure 2A). MTT (Supplemental Figure 2B) and lactate dehydrogenase (LDH) (Supplemental Figure 2C) assays showed that HG neither affected cell growth nor induced cytotoxicity. The virion productivity was not significantly affected by the 1 month–maintained cell culture process in both BHK-21 cells (Supplemental Figure 2D) and human lung epithelial A549 cells (Supplemental Figure 2E). Notably, as shown by Western blot analysis, treatment of 25 mM glucose in culture medium significantly enhanced the expression of viral NS4B protein in BHK-21 cells (*P* < 0.001) as well as in A549 cells (*P* < 0.05) under DENV infection (Figure 1A). Plaque assays further demonstrated a significant increase in virion release in 25 mM glucose medium–treated cells compared with 5.5 mM glucose medium–treated cells (*P* < 0.001) and 25 mM mannose medium–treated cells (*P* < 0.01) (Figure 1B), although a minor enhancement in virion release was also observed in the 25 mM mannose–treated, DENV-infected BHK-21 cells, suggesting the significant enhancing effect of glucose stress. In addition to BHK-21 cells, plaque assays of DENV-infected A549 cells also showed that 25 mM glucose stimulation significantly (*P* < 0.01) enhanced viral particle production (Figure 1C). The data show that HG promotes DENV infection in vitro.

### HG treatment does not affect innate responses, viral binding or entry, and viral genome replication.

Type 1 IFN is crucial to restrict viral infections (30, 31). Western blotting showed that HG resulted in comparable protein expression of phosphorylated interferon regulatory factor 3 (p-IRF3)/IRF3 in BHK-21 cells (Supplemental Figure 3A) and phosphorylated signal transducer and activator of transcription 1 (p-STAT1)/STAT1 in A549 cells (Supplemental Figure 3B). These results suggest that HG did not alter the host antiviral responses to affect virus infection in the host cells.

**Figure 2:**
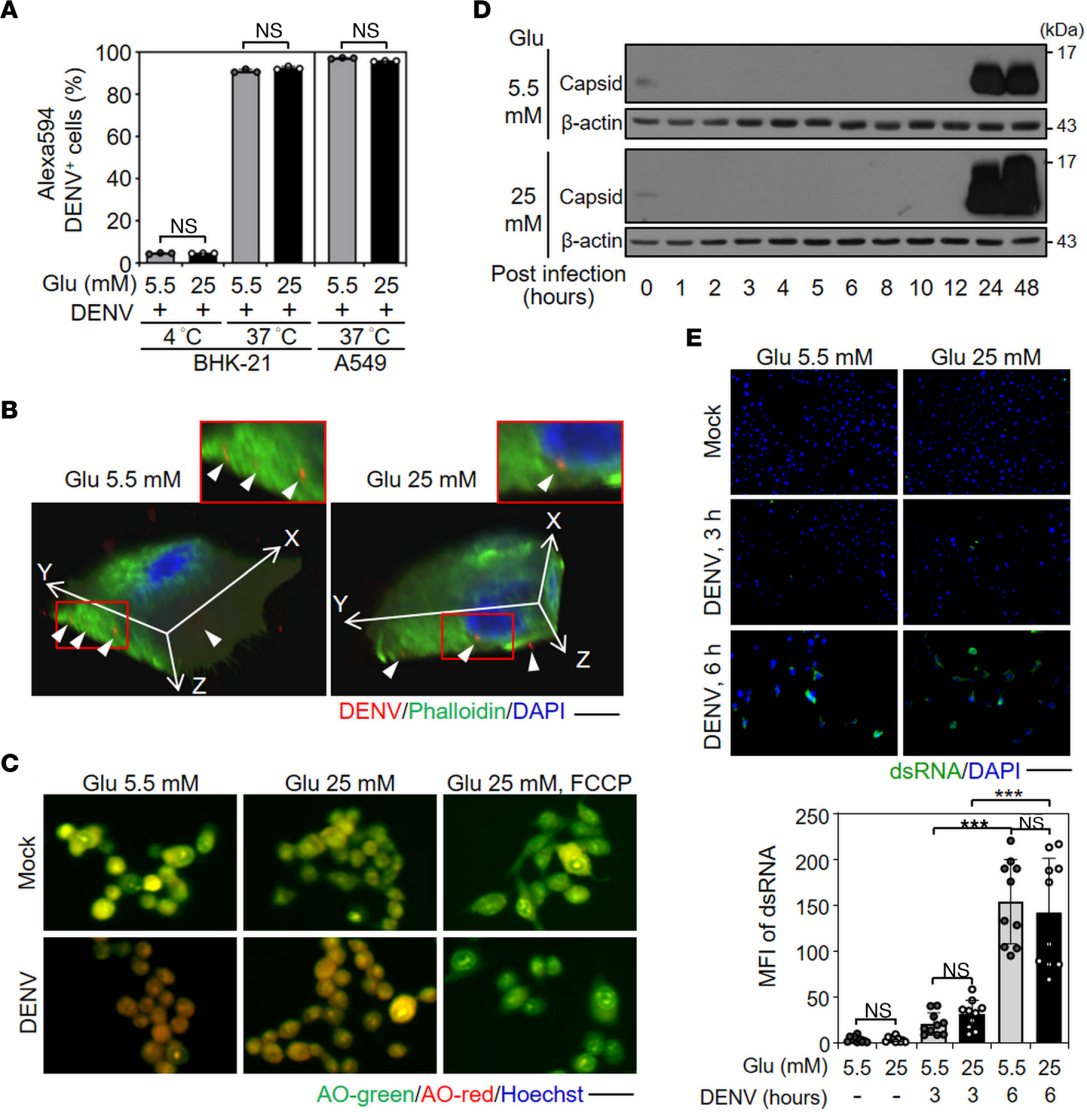
HG treatment does not significantly affect the early stage of DENV infection. BHK-21 and A549 cells were treated with 5.5 or 25 mM Glu-containing medium for 48 hours and then infected with DENV 2 for 2 hours. (**A**) Flow cytometry determined the binding (at 4°C and 0 h.p.i.) and entry (at 37°C and 2 h.p.i.) of Alexa Fluor 594–conjugated DENV 2 to cells (MOI, 10). (**B**) Confocal microscopy showed Alexa Fluor 594–conjugated DENV 2 (red) in infected BHK-21 cells (at 37°C and h.p.i.). Phalloidin (green) and DAPI (blue) staining indicated the actin filaments and nuclei, respectively. Scale bar: 20 μm. (**C**) Fluorescent images of acridine orange (AO) staining in infected BHK-21 cells (at 37°C and 2 h.p.i.). Scale bar: 100 μm. (**D**) Western blot analysis showed the expression of the DENV 2 viral capsid protein in a time-kinetic manner. (**E**) IHC images showed the viral dsRNA (green) in infected BHK-21 cells. Scale bar: 200 μm. The quantitative MFI also is shown. The individual data points indicated the MFI ratio of viral dsRNA-positive area (green) to DAPI counts (blue) from at least 3 random areas under microscopy observation. The mean ± SD of quantitative data from at least 3 independent experiments are reported. ***P < 0.001.

Flow cytometry analysis of fluorescence-labeled DENV in BHK-21, as well as A549, cells showed that neither viral binding (infection at 4°C) nor viral entry (infection at 37°C) was affected by 5.5 or 25 mM glucose-containing culture medium (Figure 2A). A single cell image obtained from confocal microscopy further showed that DENV could infect both 5.5 and 25 mM glucose medium–treated cells (Figure 2B). These results indicate that HG has no effects on DENV binding and entry.

After binding to the cell surface receptor, DENV infects host cells through endocytosis. Then, DENV conducts viral capsid uncoating, viral genome release to the cytosol, viral RNA replication, viral translation, and viral protein pr-M cleavage. Finally, the mature DENV virion is packaged and released from cells (18). Considering that HG treatment elevated V-ATPase activity (32), a key enzyme required for endosomal acidification, fluorescent microscopy analysis of acridine orange staining showed no difference in endosomal acidification in 5.5 or 25 mM glucose medium–treated BHK-21 cells (Figure 2C). Protonophore carbonyl cyanide-*p*-trifluoromethoxyphenylhydrazone–treated (FCCP–treated) cells served as a control to suppress endosomal acidification (33). The results indicate that HG does not affect endosomal acidification, suggesting that HG is unable to alter the early stages of DENV infection.

To investigate the effects of HG on the uncoating process during DENV infection, cells were infected with DENV at 4°C for 2 hours for viral binding or incubated at 37°C for an additional 2 hours for viral entry. Time course samples were harvested and analyzed by Western blotting (Figure 2D). Our results showed an identical manner of capsid protein expression in both 5.5 and 25 mM glucose medium–treated BHK-21 cells. According to the results, capsid proteins could be detected at 0 hours postinfection (h.p.i.); however, there was no detectable protein expression until 24 to 48 h.p.i., suggesting no effect of HG on DENV uncoating processes. Therefore, we further examined viral replication by immunofluorescence staining of dsRNA (Figure 2E). Viral dsRNA expression could be detected at 3 h.p.i. and was significantly (*P* < 0.001) increased at 6 h.p.i. in both the 5.5 and 25 mM glucose medium–treated BHK-21 cells. These results indicate that HG has no striking effects on host innate antiviral responses and causes fewer effects during the early steps of viral infection.

### HG promotes DENV viral translation.

**Figure 3:**
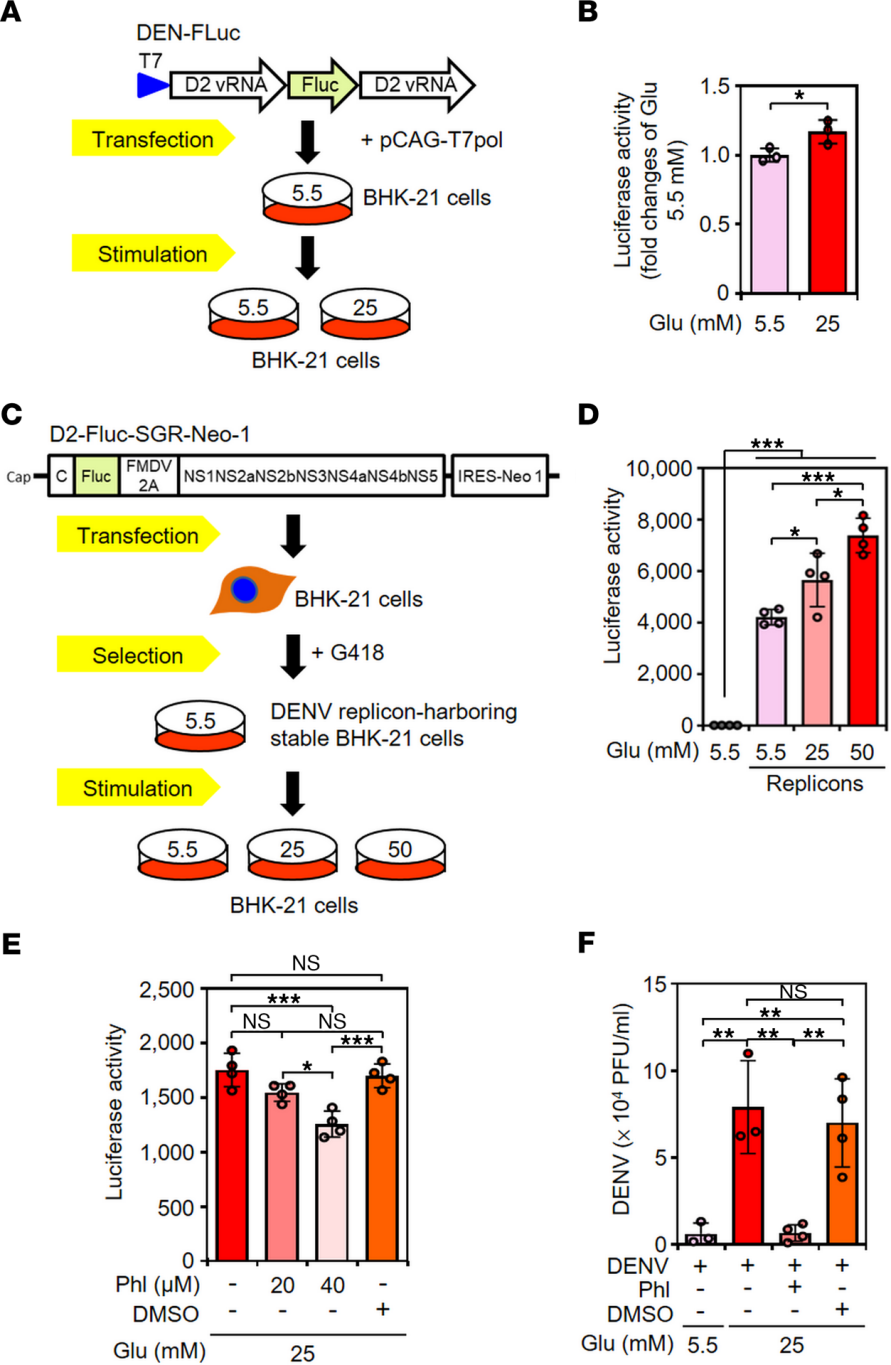
Inhibiting glucose uptake reduces HG-enhanced viral translation and virion production. (**A**) Glucose (Glu) 5.5 mM medium–maintained BHK-21 cells were cotransfected with DENV-FLuc and pCAG-T7pol after stimulation with medium containing Glu 5.5 or 25 mM for 48 hours. (**B**) Luciferase activity of DENV-FLuc–containing BHK-21 cells were detected and analyzed. (**C**) BHK-21 cells were transfected with D2-Fluc-SGR-Neo-1 and selected by G418 addition. (**D**) The luciferase activity assay showed translation activity in parental BHK-21 and BHK-D2-Fluc-SGR-Neo-1 cells (replicons) treated with the indicated concentrations of Glu for 48 hours. (**E**) Luciferase activity of replicons was determined with or without Phl treatment for 48 hours. (**F**) Plaque assays were conducted to determine the viral titer in DENV-infected BHK-21 cells treated with Glu and/or 40 μM Phl. DMSO was used as a control. The mean ± SD of quantitative data from at least 3 independent experiments are reported. *P < 0.05, **P < 0.01, ***P < 0.001.

Because HG stimulation did not affect the early viral infection steps from binding and entry to genome replication, we next assessed whether HG increases viral translation. Applying the DENV–firefly luciferase (FLuc) assay, which mimics DENV viral RNA translation (Figure 3A), the luciferase activity of BHK-21 cells containing DENV-FLuc was significantly (*P* < 0.05) increased by 25 mM glucose-medium stimulation (Figure 3B). Furthermore, the luciferase activity of D2-Fluc-SGR-Neo 1-harbored BHK-21 cells (BHK-21-SGR cells), a cellular replicon–based reporter assay (Figure 3C) (34), showed that glucose treatment caused a significant increase in activity in a dose-dependent manner (Figure 3D). MTT and LDH assays confirmed that HG treatment did not affect cell growth (Supplemental Figure 4A) or cytotoxicity (Supplemental Figure 4B) in BHK-21-SGR cells. To block the HG-enhanced translational activity, phloretin [Phl; 2′,4′,6′-trihydroxy-3-(4-hydroxyphenyl)-propiophenone], a glucose transporter blocker (35), was used in this study. Phl treatment significantly (*P* < 0.05) reduced the luciferase activity in HG-stimulated BHK-21-SGR cells (Figure 3E). Plaque assays further confirmed the significant blockade effect of Phl treatment on HG-enhanced virion release in BHK-21 cells (*P* < 0.001) (Figure 3F). Thus, HG treatment increases translation, which enhances viral production.

### HG increases viral translation by enhancing the translational factor PABP to promote DENV infection.

**Figure 4:**
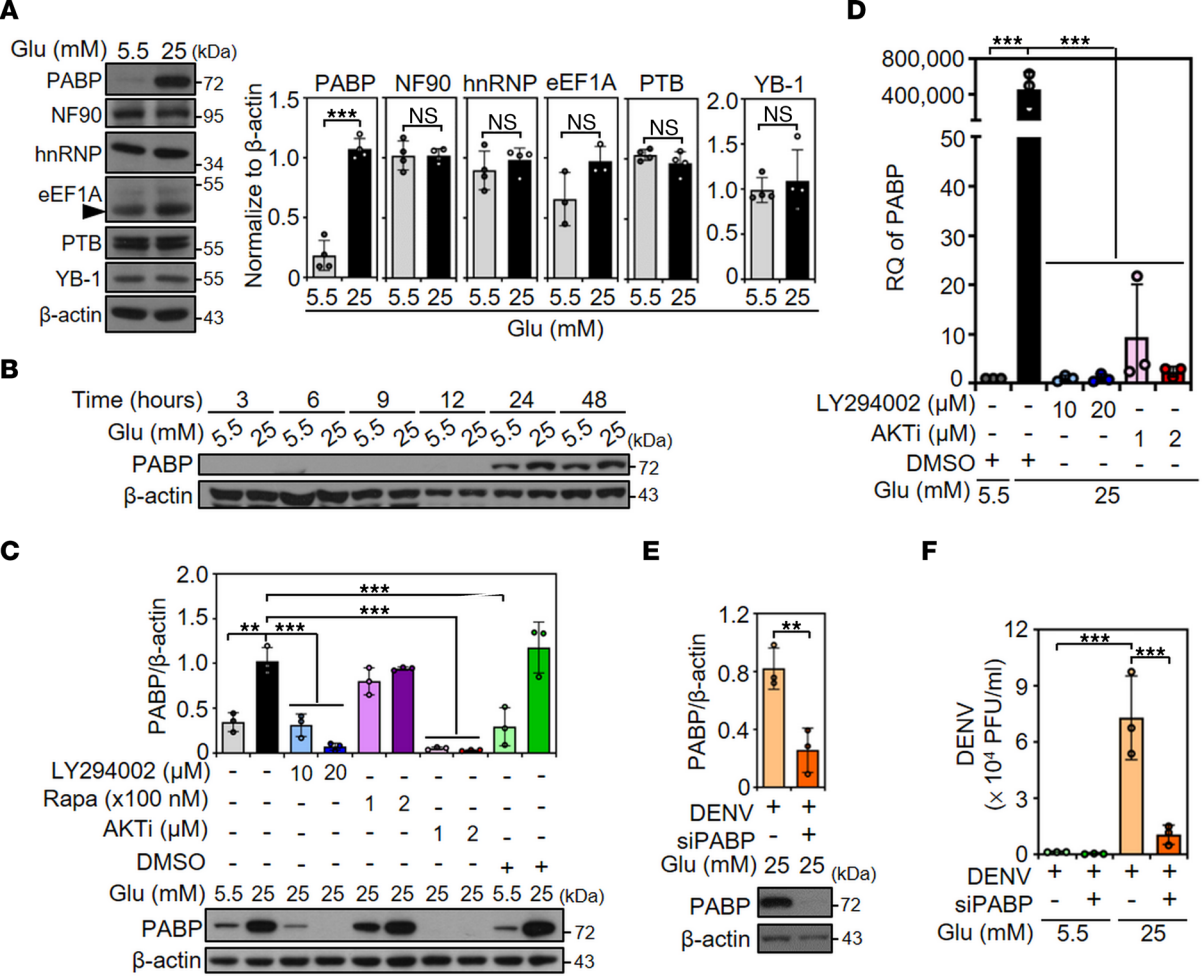
PI3K/AKT signaling contributes to HG-induced PABP expression, which promotes DENV infection. (**A**) Western blot showed the expression of PABP, NF90, hnRNP, eEF1A, PTB, YB-1, and β-actin in 5.5 or 25 mM glucose (Glu) medium–treated BHK-21 cells for 48 hours. (**B**) Furthermore, the time course expression of PABP protein also is shown. (**C**) Western blot showed PABP protein expression in BHK-21 cells that were pretreated with or without PI3K inhibitor (LY294002), the mTOR inhibitor rapamycin (Rapa), or AKT inhibitor (AKTi) for 1 hour followed by 5.5 or 25 mM Glu-containing–medium treatment for 48 hours. (**D**) Real-time qPCR assays showed the expression of PABP mRNA in 5.5 or 25 mM Glu-treated BHK-21 cells that were pretreated with or without LY294002 and an AKTi for 1 hour and subsequently maintained in medium containing 5.5 or 25 mM Glu for 48 hours. (**E**) Western blot showed PABP protein expression in BHK-21 cells pretreated with PABP siRNA (siPABP) for 48 hours, followed by incubation with medium containing 25 mM Glu. Cells without control siRNA pretreatment were used as negative control. (**F**) Plaque assays were conducted to determine the viral titer of BHK-21 cells that were pretreated with PABP siRNA for 48 hours and then infected with DENV 2 (MOI, 1) for an additional 48 hours in 5.5 or 25 mM Glu-containing medium. DMSO was used as a control. The mean ± SD of quantitative data from at least 3 independent experiments are reported. **P < 0.01, ***P < 0.001. RQ, relative quantification.

To explore the possible mechanisms of how HG promotes viral translation, we investigated the involvement of host factors that are reported to contribute to DENV translation, including PABP, NF90, hnRNP, eEF1A, PTB, and YB-1 (36). Western blot analysis showed that only PABP expression was significantly increased (*P* < 0.001) in 25 mM glucose medium–treated BHK-21 cells (Figure 4A) and A549 cells (Supplemental Figure 5). A time course analysis of PABP protein expression was also demonstrated by Western blotting (Figure 4B).

To investigate the regulation of PABP induction, the PI3K/AKT/mTOR signaling cascade was targeted regarding its roles in regulating cell metabolism (37, 38). As shown by Western blot analysis, treatment with the PI3K inhibitor LY294002 and an AKT inhibitor, but not the mTOR inhibitor rapamycin, significantly (*P* < 0.05) blocked PABP expression in 25 mM glucose medium–treated BHK-21 cells (Figure 4C). The results of qPCR also showed elevated relative quantification of PABP mRNA, which was significantly (*P* < 0.001) reduced by either the PI3K or AKT inhibitor in 25 mM glucose medium–treated BHK-21 cells (Figure 4D).

To further determine the critical role of PABP in DENV infection, an siRNA-based approach was used to knock down PABP, as shown by Western blot analysis (Figure 4E). Plaque assays showed notable inhibition of virion release in 25 mM glucose medium–treated BHK-21 cells with PABP siRNA (siPABP) (Figure 4F). Together, these results demonstrate that HG promotes PABP expression to enhance viral translation for DENV infection.

### Increased PABP-facilitated viral translation requires PDI.

**Figure 5:**
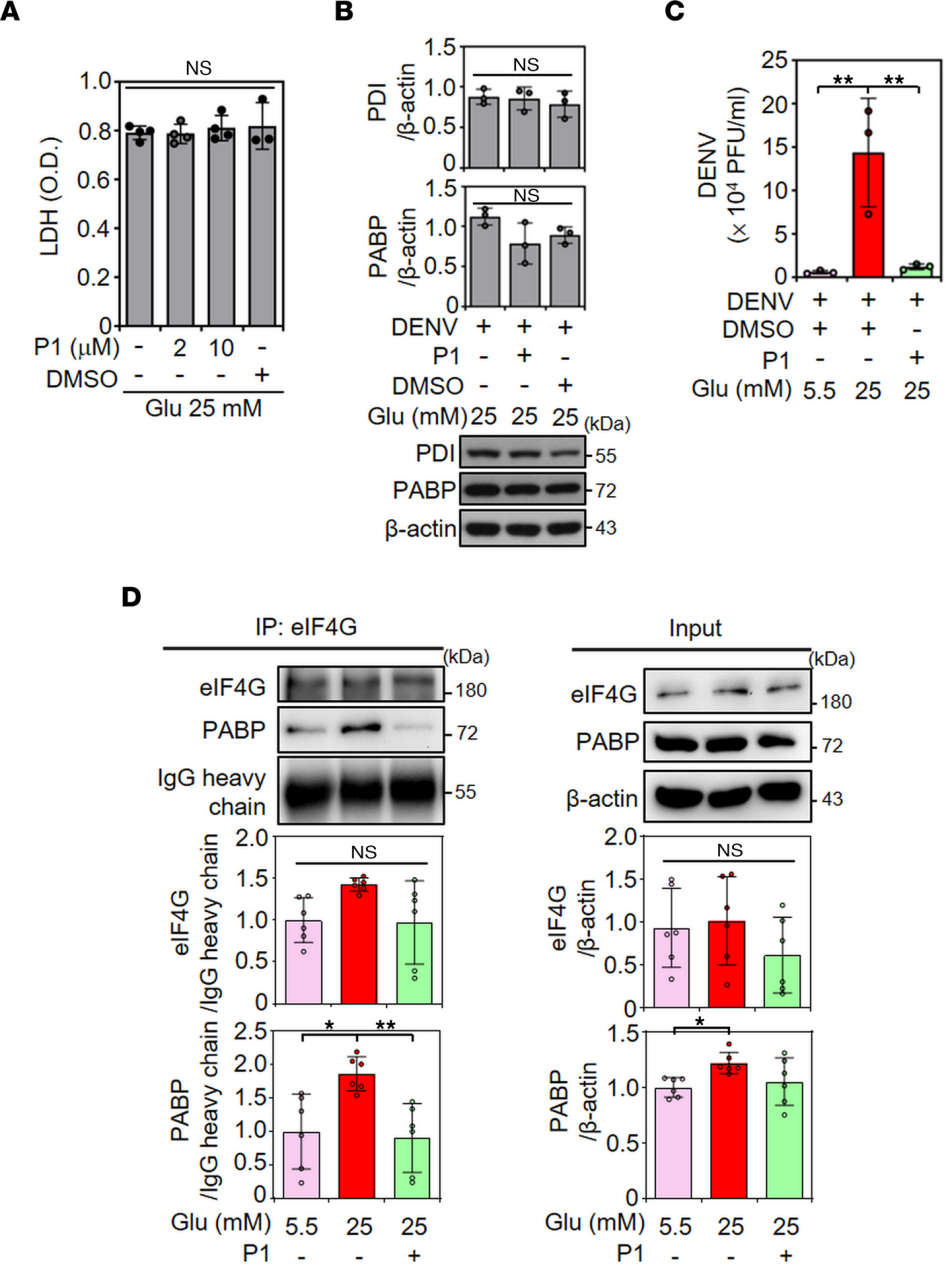
PABP-facilitated viral translation requires PDI-mediated, PABP-eIF4G translational complex formation. (**A**) The LDH assay showed cell cytotoxicity in BHK-21 cells cultured in medium containing 25 mM glucose (Glu) with or without P1 cotreatment for 48 hours. O.D., optical density. (**B**) Representative Western blot showed the protein expression of PABP and PDI in BHK-21 cells pretreated with P1 for 3 hours, followed by DENV 2 (MOI, 1) infection for an additional 48 hours. (**C**) Plaque assays were conducted to determine the viral titer of BHK-21 cells that were pretreated with P1 for 3 hours and then infected with DENV 2 (MOI, 1) for an additional 48 hours. DMSO was used as a control. (**D**) BHK-21 cells were pretreated with or without P1 for 3 hours and then further cultured in medium containing 25 mM Glu for 48 hours. Cell lysates were immunoprecipitated (IP) with anti–eIF4G Ab and then analyzed by Western blotting. The inputs represented the original cell lysates. The mean ± SD of quantitative data from at least 3 independent experiments are reported. *P < 0.05. **P < 0.01.

PDI is a crucial regulator of DENV infection by interacting with DENV NS1 in a viral translational complex, promoting DENV replication and infection (39, 40). Interestingly, under HG, PABP requires PDI to form an RNA-binding complex to promote insulin mRNA translation in β cells (29). To explore the role of PDI in PABP-modulated viral infection, we used P1, a cell-permeable small-molecule PDI inhibitor, to inhibit PDI activity (41). P1 treatment at a dose of 10 μM did not induce cytotoxicity, which was shown by LDH assay (Figure 5A). Notably, P1 treatment did not reduce PDI and PABP protein expression (Figure 5B) but significantly (*P* < 0.01) decreased virion release in 25 mM glucose medium–treated BHK-21 cells under DENV infection (Figure 5C). Coimmunoprecipitation demonstrated an increased interaction between eIF4G and PABP, which was reduced by P1 treatment in 25 mM glucose medium–treated BHK-21 cells (Figure 5D). These findings show that PDI, at least in part, contributes to the formation of the PABP-eIF4G translational complex as well as DENV replication.

### Hyperglycemia exacerbates mortality and viral replication in DENV-infected mice.

To mimic hyperglycemia in vivo, streptozotocin (STZ), a compound that is toxic to pancreatic islet β cells, was administered 3 times by i.p. injection to immunocompetent, outbred Institute of Cancer Research (ICR) mice (Supplemental Figure 6A) to induce diabetic hyperglycemia, as monitored by blood sugar levels. The results showed higher blood sugar levels in STZ-injected mice than in vehicle-injected mice (Supplemental Figure 6B). We next verified the hyperglycemic effect on PABP expression as well as DENV infection in vivo using several types of cell lysate. Using Western blot analysis (Supplemental Figure 6C), there was no remarkable difference in PABP protein expression in the heart, lung, spleen, kidney, brain, and spinal cord between vehicle-treated and STZ-treated mice. In these organs and tissues, no DENV NS1 viral protein was detected. However, the expression of viral NS1 protein showed an increasing trend, whereas there was no significant difference in the protein expression of PABP in the liver of STZ-treated mice (Supplemental Figure 6D). These data indicate the enhanced effect of diabetic hyperglycemia on DENV infection in vivo.

**Figure 6:**
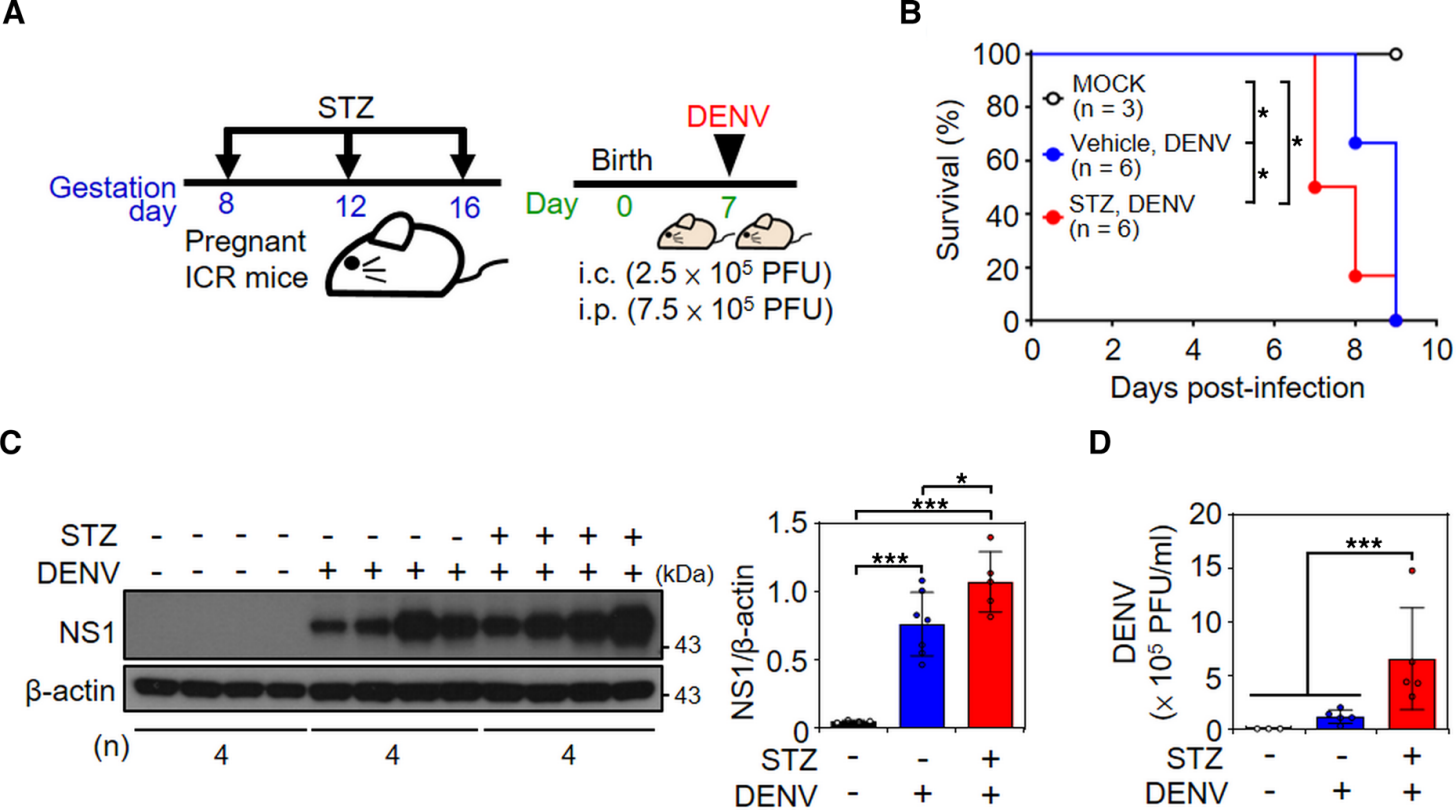
STZ-induced hyperglycemic mice had increased mortality rate and viral replication under DENV infection. (**A**) Pregnant ICR mice were i.p. injected 3 times with vehicle or STZ, as indicated. Seven-day-old ICR suckling mice were concurrently intracranially (i.c.) and i.p. inoculated with DENV 2. (**B**) The time-kinetic changes in the survival rates of the mice were monitored. (**C**) Western blot analysis showed viral NS1 protein expression in the brain of mice at 8 days postinfection. (**D**) Plaque assays were conducted to determine the viral titer. Each data point represents 1 mouse. The survival rate followed a log-rank test and the values are presented as the mean ± SD. The mean ± SD of quantitative data from at least 3 independent experiments are reported. *P < 0.05, ***P < 0.001.

To further investigate the effects of hyperglycemic stress on DENV infection in vivo and on viral pathogenesis, we used a murine model of DENV infection that can be injected into the mouse brain to induce encephalitis-like symptoms and death (42–46). STZ was i.p. injected into pregnant ICR mice to produce a hyperglycemic environment in mice. After birth, 7-day-old suckling mice were concurrently inoculated with DENV serotype 2 (DENV 2) by intracranial and i.p. injection (Figure 6A). Although the blood sugar level (Supplemental Figure 7A) was not remarkably difference between pups delivered by vehicle- and STZ-treated mice, the liver PABP expression (Supplemental Figure 7B) levels of 1-day-old suckling mice bred from STZ-treated mice were higher (*P* < 0.05) than those in corresponding mice without STZ stimulation.

Monitoring the survival rates showed that hyperglycemic mice had a 50% death rate at 7 days postinfection compared with the nonhyperglycemic mice (*P* < 0.05) (Figure 6B). Moreover, viral NS1 protein expression (Figure 6C) and virion production (Figure 6D) were significantly (*P* < 0.05) increased in the hyperglycemic mice infected with DENV. These results indicate that hyperglycemic stress exacerbates DENV infection by promoting viral replication, subsequently inducing mouse death.

## Discussion

DM is a chronic inflammatory disease that affects millions of people worldwide. Hyperglycemia shapes hyperpermeability in vessel systems and impairs immune responses, which are considered possible reasons for the progression of severe dengue disease in patients with DM. Here, we revealed a molecular mechanism whereby HG promotes DENV infection. According to our findings, HG treatment had no effects on viral attachment or entry and on antiviral IFN responses. Still, HG treatment enhanced viral titer and viral protein expression by promoting the host translational factor PABP. Additional therapeutic strategies that target HG-induced PABP through blockade of PI3K/AKT signaling and direct knockdown of PABP by inhibiting glucose uptake and interrupting translational complex formation could reduce DENV replication. DM-conditioned mice also had a higher mortality rate, viral protein expression, and brain viral loads. These results indicate that hyperglycemic stress promotes the infectivity of DENV by facilitating viral translation.

We showed that HG stimulation increased the mRNA level of PABP (Figure 4D); however, the transcriptional enhancement by HG stimulation is still unclear. The adenine-rich PABP 5′-UTR serves as a repressive autoregulatory sequence to inhibit PABP expression (47). In response to growth and nutritional stimulation, the terminal oligopyrimidine-tract motif in the PABP 5′-UTR can mediate its translational control (48), indicating the self-regulation of PABP translation. For transcriptional regulation, the chromosomal location of PABPC1 is 8q22.2-q23 (49), where the top transcription factor binding sites by QIAGEN in the PABPC1 gene promoter are AP-2α, AP-2αA, AP-2β, AP-2γ, Brachyury, E47, Elk-1, HFH-1, Pax-5, and TBP. Our microarray analysis data showed the expression of these predicted transcriptional factors is mostly upregulated in HG-treated A549 cells (Supplemental Table 2), suggesting that HG enhances PABP by potentially promoting these relevant transcription factors of PABP.

The PI3K/AKT/mTOR pathway may modulate PABP expression under environmental stimulation via growth factors, hormones, and cytokines (37). The PI3K/AKT pathway has been reported to be triggered by DENV to maintain the survival of infected cells, which could be inhibited by AR-12, a celecoxib derivative that suppresses PI3K/AKT signaling and GRP78 expression to limit DENV replication (50, 51). Consistently, our data also showed that blockade of PI3K/AKT could abolish HG-induced PABP mRNA and protein expression (Figure 4, C and D). Therefore, these findings provide further evidence of the PI3K/AKT/PABP–mediated amplification of DENV replication and a possible strategy to inhibit DENV replication by targeting HG-induced PI3K/AKT/PABP axis signaling.

During infection, type 1 IFNs could be induced through the recognition of viral RNA by the host pattern-recognition receptors TLR3, RIG-I, and MDA-5. The IFN-triggered antiviral responses could eliminate early viral replication and spread (52, 53). In addition to their immune roles, type 1 IFNs have been shown to inhibit DENV by blocking viral translation in a protein kinase R–independent pathway (54). Suzuki et al. (55) further revealed that *C19orf66*, an IFN-stimulated gene, inhibits DENV replication by blocking PABP-mediated translation, indicating the crucial role of PABP in promoting DENV translation. Balinsky et al. (56) also found that C19orf66 is upregulated after DENV infection in an IFN-dependent manner. Moreover, they found that the RNA-binding protein C19orf66 associates with the DENV replication complex and colocalizes with P bodies, sites where RNAs decay (56). Although HG treatment did not alter the type 1 IFN–related signaling molecules p-STAT1 and p-IRF3 (Supplemental Figure 3, A and B), we found that exogenous IFN-α treatment markedly inhibited the luciferase activity of HG-treated BHK-21-SGR replicons (Supplemental Figure 8), indicating that type 1 IFNs effectively abolish viral translation, probably by targeting PABP. Collectively, IFN-induced C19orf66 inhibits DENV replication by potentially abrogating PABP-mediated viral translation and also by hijacking DENV and PABP to the P bodies for degrading viral RNA.

Phl, a natural phenol that belongs to the chalcone class of flavonoids, is currently considered a DM therapy because of its action on glucose transporter regulation. In the human triple-negative breast cancer cell line MDA-MB-231, Phl directly targets GLUT2, which results in the deprivation of glucose uptake (57). Phl is also a glucose transporter blocker similar to other glucose antagonists, including quercetin, WZB117, and STF31. Inhibiting extracellular glucose uptake reduces glycolysis, which is characterized by decreased lactate production and increased cell apoptosis (58). Another anti–type 2 DM drug, metformin, has also been reported to reduce hepatic gluconeogenesis and has been used, therefore, as a joint antihyperglycemic agent in recent decades (59). Importantly, a clinical observation study showed that the use of metformin reduces the risk of developing severe dengue diseases; it has been hypothesized that metformin reduces the risk of severe bleeding in DENV-infected patients (60). According to our findings in the present study, the antidiabetic agent Phl can use as a potential therapy for DENV infection because treatment with the compound shows significant inhibition of DENV replication by targeting the glucose regulator.

In conclusion, this study provides a possible mechanism by which HG promotes severe DENV infection by potentially inducing PABP to facilitate viral translation. Targeting PABP and the translational complex or inhibiting glucose uptake can reduce viral replication. PABP also plays a pivotal role in many viral infections, such as influenza virus, rotavirus, and human CMV (61). This evidence raises caution that infections may be exacerbated by an abundance of PABP in an HG environment, which requires further exploration. Overall, this study reveals the pathogenic role of HG-induced PABP in DENV infection and further demonstrates a potential antiviral strategy of inhibiting PABP-mediated viral replication.

## Methods

### Ethics statement and data collection.

For the epidemiological analysis, the data from 31,270 DENV-infected participants and 123,776 participants who had no DENV disease diagnosis were obtained from the NHIRD according to the records from the Taiwan CDC. Briefly, suspected dengue cases were confirmed by the detection of anti-dengue IgM, viral nucleotide sequence, virion presence, or DENV NS1 antigen. The dengue disease severity was categorized as DF or DHF, on the basis of World Health Organization 1997 criteria (62). The control groups were collected and processed by the NHIRD and Department of Statistics, Ministry of Health and Welfare. The DM group contained patients with type 1 DM and patients with type 2 DM. Those with type 1 DM were included according to the Registry for Catastrophic Illness Patient Database. Those with type 2 DM were defined as outpatient department patients who had confirmed the type 2 DM diagnosis at 2 visits within 6 months (every visit interval time was more than 30 days).

### Cell, virus, and mice.

The BHK-21 fibroblasts (ATCC,CCL10) and human lung epithelial A549 cells (ATCC CCL185) were cultured in DMEM (Thermo Fisher Scientific) containing 5.5 mM glucose (11 mmol/L), 10% heat-inactivated FBS (Biological Industries), 1% penicillin–streptomycin (Thermo Fisher Scientific), and 1% sodium pyruvate (Thermo Fisher Scientific) at 37°C in 5% CO_2_. For HG, cells were cultured in DMEM containing 25 mM (50 mmol/L) glucose, 10% heat-inactivated FBS, 1% penicillin–streptomycin, and 1% sodium pyruvate at 37°C in 5% CO_2_. BHK-21 cells harboring a luciferase-expressing DENV replicon (BHK-D2-Fluc-SGR-Neo-1) were maintained in DMEM containing 5.5 mM glucose (11 mmol/L), 10% heat-inactivated FBS, 1% penicillin–streptomycin, 1% sodium pyruvate, and 0.4 mg/mL G418 agent (MilliporeSigma) at 37°C in 5% CO_2_. *A*. *albopictus* clone mosquito C6/36 cells (ATCC, catalog CRL1660) were maintained in MEM (Thermo Fisher Scientific) containing 10% heat-inactivated FBS, 1% penicillin–streptomycin, 1% sodium pyruvate, 1% HEPES (Thermo Fisher Scientific), and 1% nonessential amino acids (Thermo Fisher Scientific) at 28°C in 5% CO_2_. The monolayer of C6/36 cells was infected with DENV serotype 2 (strain PL046, from Taiwan CDC) at an MOI of 0.01 and incubated at 28°C in 5% CO_2_ for 5 days. The viral supernatants were collected and filtered with a 0.22 μm filter followed by storage at –80°C until use. Viral titer was determined by plaque assay using BHK-21 cell monolayers. Alexa Fluor 594 NHS ester–conjugated (Molecular Probes) DENV particles were prepared according to a previous study (63). ICR mice were purchased from BioLASCO Taiwan.

### Abs and reagents.

Abs against eEF1A1(catalog GTX102285), PDI (clone RL77; catalog GTX25484), DENV NS1(catalog GTX124280), NS4B (catalog GTX103349), and capsid (catalog GTX103343) were purchased from GeneTex. Abs against PABP (clone 10E10; catalog NB120-6125; Novus Biologicals); dsRNA (catalog 10010200; SCICONS); phospho-IRF3^Ser396^ (catalog bs-3195R; Bioss); β-actin (catalog A5441; MilliporeSigma); IRF3 (clone D83B9; catalog 4302), phospho-STAT1^Tyr701^ (clone 58D6; catalog 9167), STAT1 (catalog 9172), HRP-conjugated goat anti–rabbit IgG (catalog 7074S), and HRP-conjugated horse anti–mouse IgG (catalog 7076S) were purchased from Cell Signaling Technology. Abs against hnRNP A2B1 (catalog ab24409) and eIF4G1 (catalog ab2609) were purchased from Abcam. Abs against YB-1 (clone A-16; catalog sc-18057), NF90 (clone N-18; catalog sc-22530-R), and HRP-conjugated donkey anti–goat IgG (catalog sc-2020) were purchased from Santa Cruz Biotechnology. Abs against PTBP1 (clone 1; catalog 32-4800), mouse IgG2b κ isotype control (clone eBMG2b; catalog 14-4732-85), and Alexa Flour 488–conjugated goat anti–mouse IgG (catalog A-11029) were purchased from Thermo Fisher Scientific. DMSO (catalog AD0470; Bionovas); PI3K inhibitor 2-(4-morpholinyl)-8-phenyl-4H-1-benzopyran-4-one hydrochloride (LY294002; catalog 70920; Cayman Chemical); P1 (catalog 5127; Tocris Bioscience); Alexa Fluor 488–conjugated phalloidin (catalog A12379; Invitrogen); DAPI (catalog D9542), STZ (catalog S0130), FCCP (catalog C2920), acridine orange hemi(zinc chloride) salt (catalog A6014), phloretin (catalog P7912), rapamycin (catalog 553210), cycloheximide (catalog C4859), and AKT inhibitor 5-(2-benzothiazolyl)-3-ethyl-2-[2-(methylphenylamino)ethenyl]-1-phenyl-1H-benzimidazolium iodide (catalog B2311) were purchased from MilliporeSigma. MTT assay and LDH assay were conducted with thiazolyl blue tetrazolium bromide (catalog T0793; Bio Basic) and a cytotoxicity detection kit (catalog 11644793001; Roche), respectively.

### Western blotting.

Proteins from collected cells were extracted with lysis buffer containing a protease inhibitor mixture (MilliporeSigma). Extracted proteins from cell lysates were separated by SDS-PAGE followed by transfer to a PVDF membrane (MilliporeSigma). The membrane was blocked with 5% nonfat milk in Tris-buffered saline containing 0.05% Tween-20 (TBS-T) at room temperature for 1 hour. Then the membrane was washed 3 times with TBS-T buffer, followed by incubation with the indicated Abs at 4°C overnight. After 3 washes with TBS-T buffer, the membrane was incubated with the indicated HRP-conjugated secondary Abs. The Ab–protein complexes on the PVDF membrane were detected using an ECL Western blot detection kit (catalog ORT2655; PerkinElmer). The ImageJ software (Fiji Software) was used to calculate the relative densities of the identified proteins.

### Plaque assay.

BHK-21 cells were plated onto 12-well plates at 1 × 10^5^ cells per well and cultured in DMEM at 37°C in 5% CO_2_ overnight. Monolayers of BHK-21 cells were infected with a serially diluted viral solution for 2 hours, which was then replaced with fresh DMEM containing 4% FBS and 0.5% methylcellulose (catalog M0512; MilliporeSigma) for 5 days. The methylcellulose medium was removed, and the wells were washed with 2 mL of PBS per well 3 times. Then the cells were fixed and stained with crystal violet solution containing 1% crystal violet (catalog C0775; MilliporeSigma), 0.64% NaCl, and 2% formaldehyde (MilliporeSigma) overnight. Subsequently, the plates were water-washed and air-dried.

### FACS analysis.

Cells were harvested and washed twice with 2 mL of iced staining buffer (PBS plus 2% FBS plus 0.1% NaN_3_) and then immunoblocked with 5% BSA blocking buffer (MilliporeSigma) at 4°C. After 30 minutes, the cells were washed with 2 mL of iced staining buffer and fixed with 200 μL of 4% paraformaldehyde (MilliporeSigma) at room temperature for 15 minutes. After fixation and washing, the cells were immunohybridized with specific primary Abs at 4°C for 40 minutes. Then, the cells were washed with 2 mL of iced staining buffer followed by staining with target immunofluorescence-conjugated secondary Abs at 4°C for 30 minutes. The cells were washed with 2 mL of staining buffer and then resuspended in 300 μL of iced staining buffer. The resuspended cells were analyzed with a BD FACSCalibur system and the FlowJo software.

### Immunofluorescence staining.

BHK-21 cells were placed in monolayers on sterile glass slides overnight, followed by treated medium containing the indicated glucose concentration for 48 hours. Then, cells were infected with Alexa Fluor 594–conjugated DENV 2 for 2 hours. After a wash with iced PBS 3 times, cells were fixed with 4% paraformaldehyde (MilliporeSigma) at room temperature for 15 minutes. Then cells were washed 3 times with iced PBS and permeabilized with permeabilization buffer (PBS plus 1% Triton X-100) at room temperature for 5 minutes. The cells were then washed 3 times with iced PBS and immunoblocked with blocking buffer (PBS plus 1% BSA plus 0.01% Triton X-100) at 4°C. After 30 minutes, cells were washed 3 times with iced PBS and stained with Alexa Fluor 488–conjugated phalloidin (Invitrogen) as well as DAPI (MilliporeSigma) at room temperature for 15 minutes. Cells were washed with iced PBS 3 times and then visualized with the laser-scanning confocal microscope (TCS SP5 Confocal Spectral Microscope Imaging System). The pixel format and gains used of the captured field were 1024 × 1024 and 590.0V, respectively. The 3D images from a series of confocal images together with the *z*-axis of the cells and the analysis of the z-stacks were reconstructed using the Leica Confocal Software. DAPI was used for nuclear staining.

### dsRNA staining.

Cells were washed with iced PBS 3 times and fixed with 4% paraformaldehyde (MilliporeSigma) at room temperature for 15 minutes. Then the cells were washed 3 times with iced PBS and permeabilized with permeabilization buffer (PBS plus 1% Triton X-100) at room temperature for 5 minutes. The cells were then washed 3 times with iced PBS and immunoblocked with blocking buffer (PBS plus 1% BSA plus 0.01% Triton X-100) at 4°C for 30 minutes. Next, cells were washed 3 times with iced PBS and immunohybridized with mouse anti–dsRNA J2 primary Ab at 4°C overnight. Subsequently, the cells were washed 3 times with iced PBS and stained with Alexa Fluor 488–conjugated goat anti–mouse Ab (Thermo Fisher Scientific) and DAPI at room temperature for 15 minutes. The cells were washed 3 times with iced PBS and then visualized with fluorescent or confocal microscopy. DAPI was used for nuclear staining.

### Acridine orange staining.

Cells were washed with HBSS (Thermo Fisher Scientific) once, then stained with acridine orange agent (MilliporeSigma) and Hoechst 33258 (Thermo Fisher Scientific) in the incubator at 37°C in 5% CO_2_. After 45 minutes, cells were washed with HBSS once and rinsed with HBSS. Subsequently, cells were visualized with fluorescent microscopy. Hoechst 33258 was used for nuclear staining.

### DENV-FLuc assay.

A DENV-FLuc assay kit mimicking DENV viral RNA translation was developed by Chia-Yi Yu (National Institute of Infectious Diseases and Vaccinology, National Health Research Institutes, Miaoli, Taiwan). The plasmids of DENV-FLuc (3,333 bp), which contain DENV viral RNA fused with FLuc under the transcriptional control of the T7 promoter, and pCAG-T7pol (Addgene, plasmid 59926) were cotransfected into glucose 5.5 mM medium–maintained BHK-21 cells by TurboFect Transfection Reagent (catalog R0531; Thermo Fisher Scientific). Then the cells were seeded in 96-well plates at 3,000 cells/well and stimulated with DMEM containing glucose 5.5 or 25 mM at 37°C in 5% CO_2_ for 48 hours. The luciferase activity was detected using the Dual-Glo luciferase assay system (catalog E2940; Promega) according to a previous study (42).

### Reporter assay.

The BHK-21-SGR cells were provided by Huey-Nan Wu (Institute of Molecular Biology, Academia Sinica, Taipei, Taiwan). Briefly, the D2-Fluc-SGR-Neo-1 was transfected into BHK-21 cells by electroporation. By G418 addition, the DENV replicon–harboring stable BHK-21 cells were established (Figure 3C) (34). Cells were seeded in 96-well plates at 3,000 cells/well and cultured in DMEM at 37°C in 5% CO_2_ overnight. After the treatments, the luciferase activity was detected using the Dual-Glo luciferase assay system (catalog E2940; Promega) according to a previous study (42).

### RT-PCR and qPCR.

Cells were extracted using TRIzol (Invitrogen) RNA extraction reagent. According to the manufacturer’s instructions, cDNA was synthesized with an RT reaction using a High-Capacity cDNA Reverse Transcription Kit (catalog 4368814; Thermo Fisher Scientific). The concentration of cDNA was determined by spectrophotometer at 260 nm. We applied 1,000 ng/μL cDNA to conduct qPCR using 2× qPCRBIO SyGreen Mix Hi-ROX (catalog PB20.12; PCR Biosystems). The PCR was performed using a StepOnePlus real-time PCR system (Applied Biosystems) with the following pairs of specific primers: primer sequences for *PABP* (forward): 5′-CCCAGCTGCTCCTAGACC-3′ and (reverse): 5′-GAGTAGCTGCAGCGGCT-3′; for DENV *NS1* (forward): 5′-ATGAATTCACGCAGCACC-3′ and (reverse): 5′-TTATCGGGTCCTGAGCATTTC-3′; for *β-actin* (forward): 5′-TCCTGTGGCATCCACGAAACT-3′ and (reverse): 5′-GAAGCATTTGCGGTGGACGAT-3′.

### siRNA knockdown.

The chemically synthesized 21-mer PABP siRNA duplexes were purchased from Dharmacon Inc. The PABP siRNA sequences used in this study are as follows: 5′-GAAAGGAGCUCAAUGGAAAUU-3′ (sense) and 5′-UUUCCAUUGAGCUCCUUUCUU-3′ (antisense). The control siRNA Stealth RNAi siRNA Negative Control was purchased from Invitrogen (Med GC, catalog 12935). For transfection, briefly, cells were plated into 12-well plates at 1 × 10^5^ cells/well and transfected with the corresponding siRNA using the TurboFect Transfection Reagent (catalog R0531; Thermo Fisher Scientific), following the manufacturer’s instructions in 5.5 mM glucose-containing medium. Twenty-four hours after transfection, the cells were treated with a 5.5 or 25 mM glucose-containing medium for 48 hours. Then, on the basis of the different experimental designs, the cells were either collected or further infected with DENV.

### Co-immunoprecipitation.

Cell lysate (1 mg) were incubated with anti–eIF4G Ab (catalog ab2609; Abcam) and protein A agarose beads (catalog 16-156; MilliporeSigma) at 4°C for 16 hours. The pulldown samples were further subjected to Western blotting and hybridized with the anti–PABP Ab (clone 10E10; catalog NB120-6125; Novus Biologicals).

### Hyperglycemic adult mouse model.

STZ was freshly dissolved in 0.05 M citric acid buffer (pH 4.5) and used within 30 minutes. All the ICR mice were fasted for 6 hours before the injection. Then, mice of the study group were i.p. injected with STZ at a dose of 200 mg/mouse body weight (kg) every 4 days for a total of 3 injections. Mice of the control group were treated with an equal volume of citrate buffer (pH 4.5) by i.p. injection. After every injection, all mice were given 10% sucrose water for 24 hours (64, 65). The blood glucose values of the hyperglycemic group should increase to greater than 250 mg/dL. Then the mice were infected with DENV 2 (PL046) by i.p. inoculation with 1 × 10^7^ PFU/mL virus. At the indicated days, mice were sacrificed to collect samples. Organs were harvested and homogenized in PBS to determine the protein expression and tissue viral load by Western blotting and plaque assay, respectively.

### Hyperglycemic suckling-mouse model.

The same approach was applied to induce hyperglycemia-conditioned ICR mice at gestation days 8, 12, and 16. The blood glucose levels of newborn pups were measured and recorded. The DENV-infected suckling murine model was created according to our previous study (45). Briefly, 2.5 × 10^5^ PFU/mL DENV 2 (PL046) were intracranially injected into the lambda area, the point at the intersection of the sagittal and lambdoid suture, of 7-day-old ICR mice. Concomitantly, mice were i.p. infected with 7.5 × 10^5^ PFU/mL DENV 2. The mice were monitored, and the time-kinetic changes in survival rate were recorded. At the indicated days, mice were sacrificed to collect samples. Organs were harvested and homogenized in PBS to determine the protein expression and tissue viral load by Western blotting and plaque assay, respectively.

### Data availability.

Microarray data were deposited in NCBI Gene Expression Omnibus (accession number GSE215199) (Supplemental Table 2).

### Statistics.

Clinical data were collected and designed as nested case-control studies and then analyzed by conditional logistic regression. Experimental data were analyzed using GraphPad Prism (version 8.3.0). Unpaired *t* test and 1-way ANOVA (Tukey’s multiple comparisons test) were used to determine experiments involving 2 and various groups, respectively. The survival rate followed a log-rank test. Values are reported as mean ± SD. All *P* values are for 2-tailed significance tests. A *P* < 0.05 is considered statistically significant.

### Study approval.

The human research protocol was approved by the Taipei Medical University-Joint Institutional Review Board (N201602014), and the need for written informed consent was waived by the Institutional Review Board. Animal studies of this project were performed according to the Animal Protection Act of Taiwan. All protocols, according to guidelines established by the Ministry of Science and Technology, Taiwan, were approved by the Laboratory Animal Care and Use Committee of Ministry of Science and Technology (IACUC-19-018).

## Author contributions

TJS performed most of the experiments and interpreted the results. CLC performed the confocal microscopy. CFL, CLC, and TTT participated in the design and supervision of the projects. MKJ, CYY, and PCT helped with the mouse experiments, DENV stock preparation, and DENV-FLuc assay. CHB, YCY, and CWT contributed to analyzing the epidemiological data. CYL provided the epidemiological data from the Taiwan CDC. TJS and CFL designed the concept of the project and wrote the manuscript. All authors reviewed and approved the manuscript.

## Supplementary Material

Supplemental data

## Figures and Tables

**Table 1:**
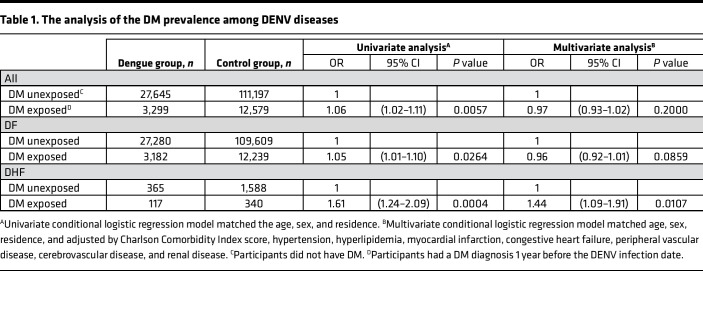
The analysis of the DM prevalence among DENV diseases

## Version 1 09/20/2022

In-Press Preview

## Version 2 11/08/2022

Electronic publication
