# Hyperglycemia exacerbates dengue virus infection by facilitating poly(A)-binding protein–mediated viral translation

**DOI:** 10.1172/jci.insight.168446

**Published:** 2023-01-24

**Authors:** Ting-Jing Shen, Chia-Ling Chen, Tsung-Ting Tsai, Ming-Kai Jhan, Chyi-Huey Bai, Yu-Chun Yen, Ching-Wen Tsai, Po-Chun Tseng, Chia-Yi Yu, Chiou-Feng Lin

Original citation: *JCI Insight*. 2022;7(21):142805. https://doi.org/10.1172/jci.insight.142805

Citation for this corrigendum: *JCI Insight*. 2023;8(2):168446. https://doi.org/10.1172/jci.insight.168446

Cheng-Yi Lee has requested to be removed from the author list, and the corresponding author has agreed. The revised author list is shown above, and the author and affiliation lists in the PDF and HTML versions have been updated accordingly.

## Version 1 01/24/2023

Electronic publication

