# Supplementary material for: Hyperglycemia exacerbates dengue virus infection by facilitating poly(A)-binding protein–mediated viral translation
Source: JCI Insight. 2022 Nov 8;7(21):e142805. doi: 10.1172/jci.insight.142805 (PMC9675471; doi:10.1172/jci.insight.142805)
Supplement: Supplemental data [file jciinsight-7-142805-s008.pdf]

Supplementary Information for

Hyperglycemia exacerbates dengue virus infection by facilitating poly(A)-binding protein-mediated viral translation

Ting-Jing Shen, Chia-Ling Chen, Tsung-Ting Tsai, Ming-Kai Jhan, Chyi-Huey Bai, Yu-Chun Yen, Ching-Wen Tsai, Cheng-Yi Lee, Po-Chun Tseng, Chia-Yi Yu, and Chiou-Feng Lin

Correspondence: cflin2014@tmu.edu.tw (C.-F. L.)

Supplemental Figures and figure legends

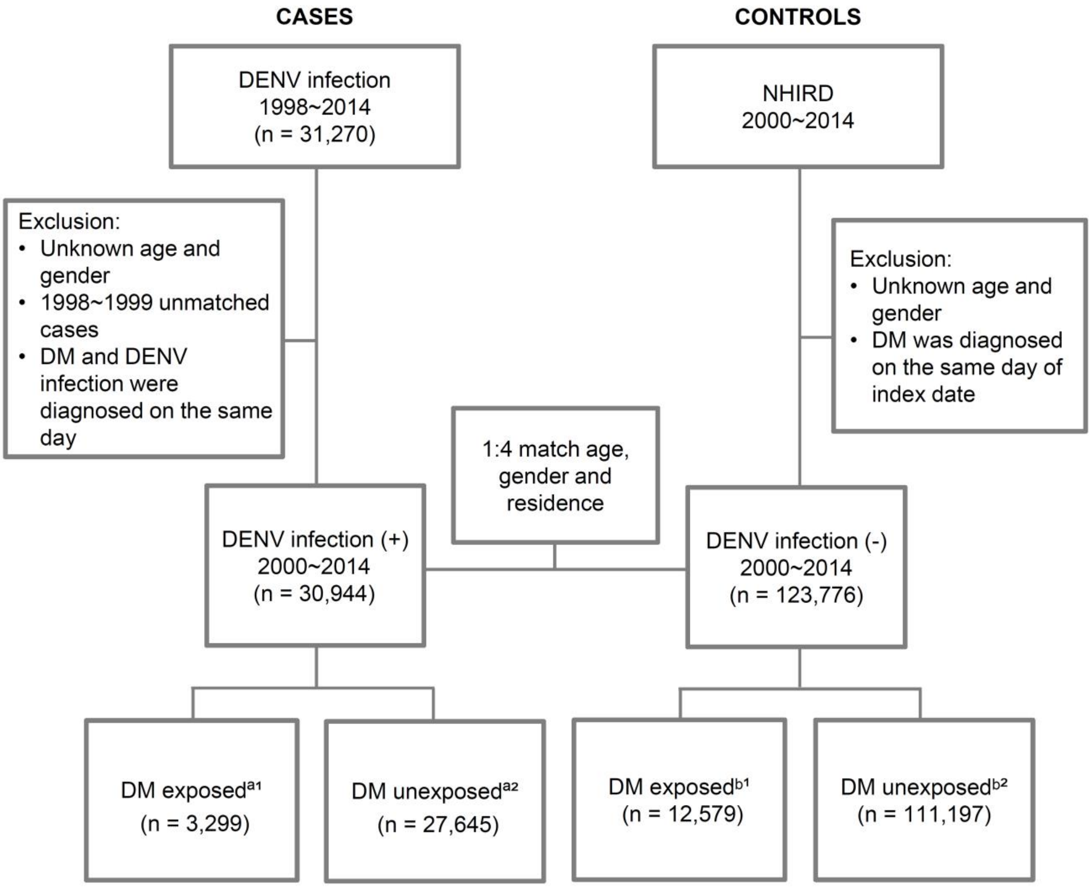

Supplemental Figure 1. Flowchart of participant selection and data process in this study. Subjects obtained from the National Health Insurance Research Database (NHIRD), depending on records from the Taiwan Centers for Disease Control (CDC) of this study were assessed and analyzed. The details of subject enrollment of this study were described in the Materials and Methods.[a1]Cases were diagnosed with DM > 1 year before who were diagnosed with DENV infection. [a2]Cases were not diagnosed with DM > 1 year before who were diagnosed with DENV infection or cases were diagnosed with DM before who were diagnosed with DENV infection within 1 year. [b1]Cases were diagnosed with DM > 1 year before the index date. [b2]Cases were not diagnosed with DM > 1 year before the index date or cases were diagnosed with DM before

the index date within 1 year. NHIRD, National Health Insurance Research Database. Index date, cases were diagnosed with DENV infection of the case group (dengue group); cases were not diagnosed with DENV infection of the control group (not dengue group).

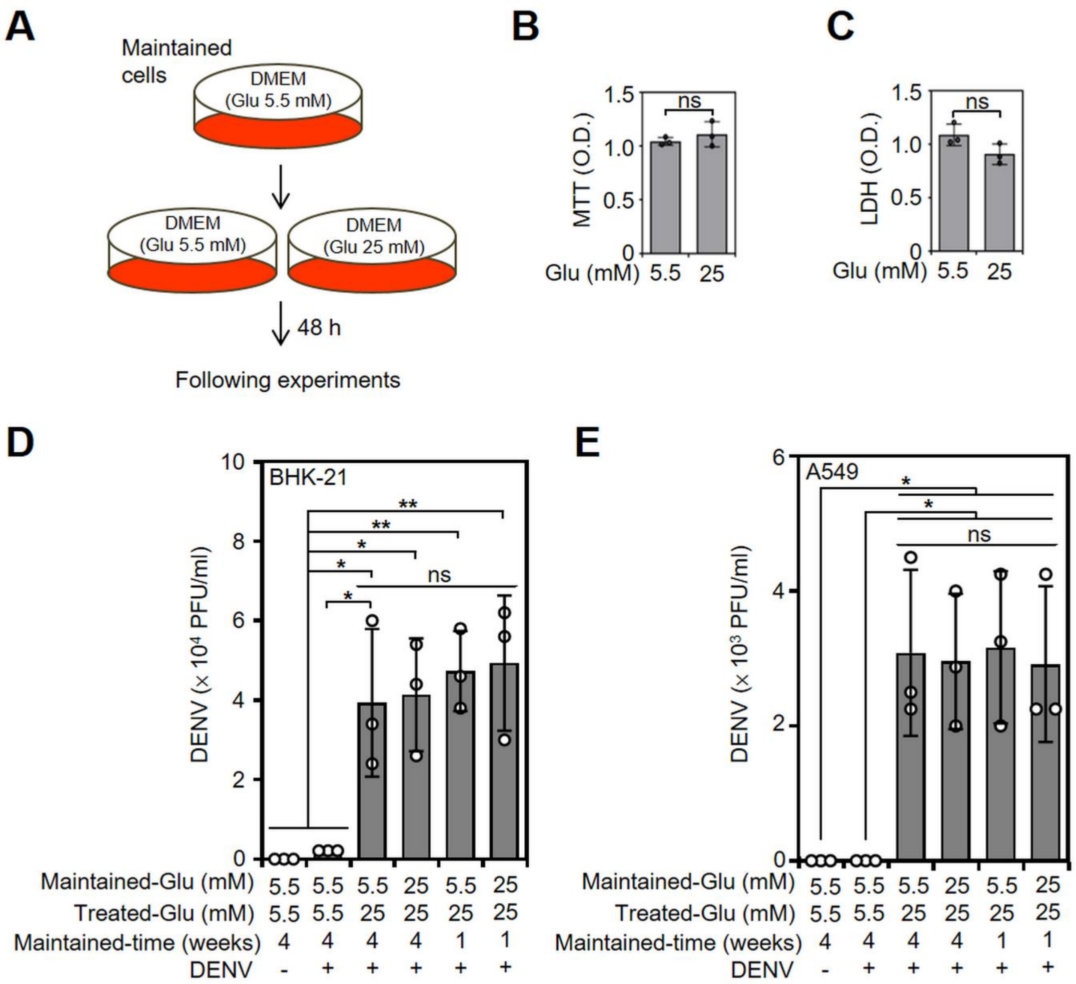

Supplemental Figure 2. HG and one-month culture process do not affect respective on cell viability and viral NS4B expression as well as virion production in BHK-21 cells. (A) Medium containing 5.5 mM glucose-maintained BHK-21 cells were treated with medium containing either 5.5 mM or 25 mM Glu for 48 hours. Then, the cells were applied to the following experiments. MTT assay **(B)** and LDH assay **(C)** represented the cell growth and cytotoxicity of one month-maintained BHK-21 cells, which were treated with medium containing 5.5 or 25 mM Glu for 48 hours, respectively. BHK-21 cells were further infected with DENV 2 (MOI = 1) for 48 hours. Plaque assay determined the viral titer of BHK-21 **(D)** and A549 cells **(E)**. Quantitative data

show the mean ± SD of three independent experiments. ns, not significant. *$P < 0.05$ and **$P < 0.01$. ns, not significant.

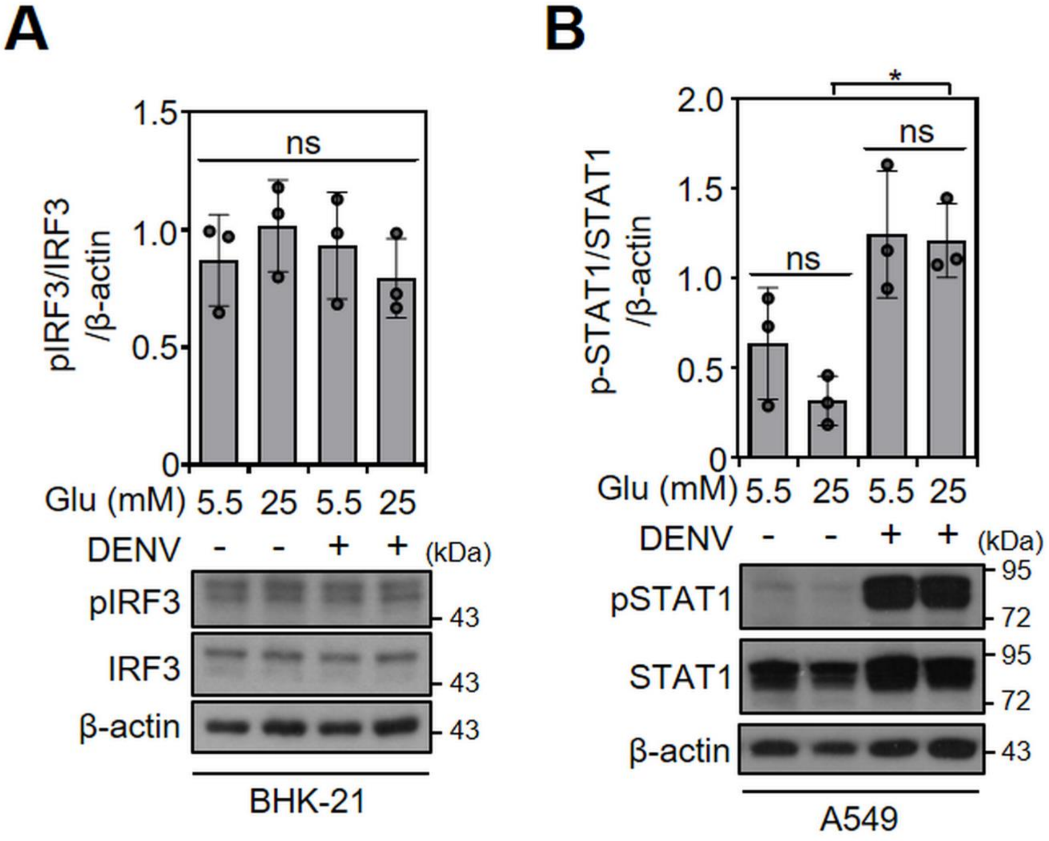

Supplemental Figure 3. HG has no remarkable effects on type 1 IFN-associated antiviral responses. Representative western blot showed the expressions of pIRF3/IRF3 and pSTAT1/STAT1 of BHK-21 **(A)** and A549 cells **(B)** 24 hours after DENV infection, respectively. Quantitative data show the mean ± SD of three independent experiments. **P* < 0.05. ns, not significant.

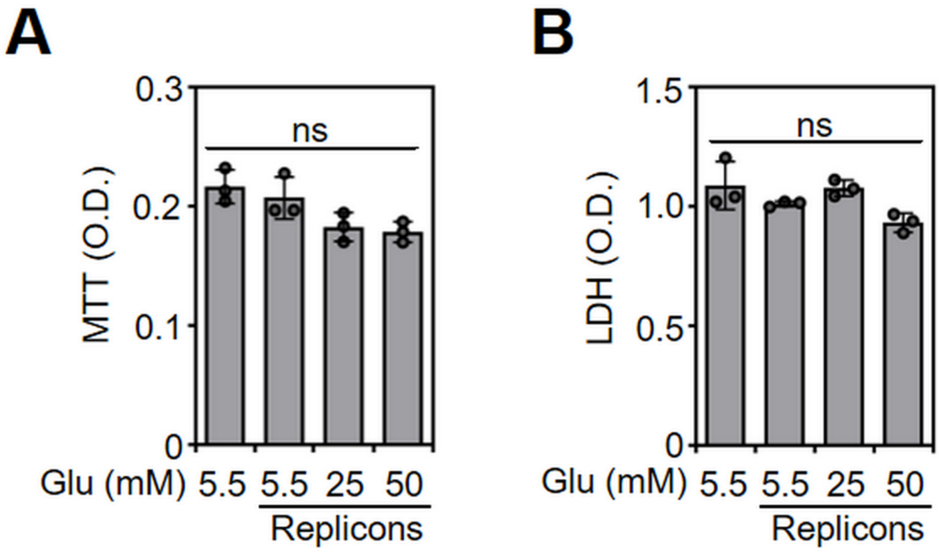

Supplemental Figure 4. HG neither affects cell growth nor induces cytotoxicity. The parental BHK-21 and BHK-D2-Fluc-SGR-Neo-1 cells (replicons) were treated with indicated concentrations of Glu for 48 hours. MTT assay **(A)** and LDH test **(B)** showed cell growth and cytotoxicity, respectively. Quantitative data show the mean ± SD of three independent experiments. ns, not significant.

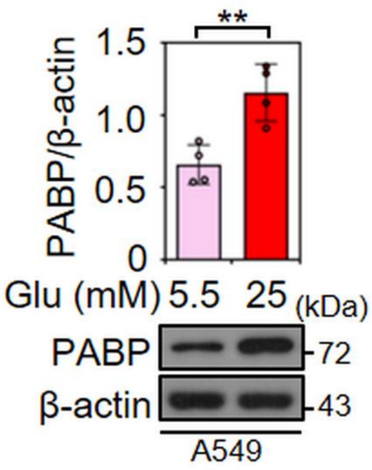

Supplemental Figure 5. HG enhances PABP expression in A549 cells. Quantitative western blot showed the expression of PABP protein expression of A549 cells, which were maintained in medium containing Glu 5.5 mM for one month then treated with the indicated concentration of Glu for 48 hours. Quantitative data show the mean ± SD of three independent experiments. $^{**}P < 0.01$.

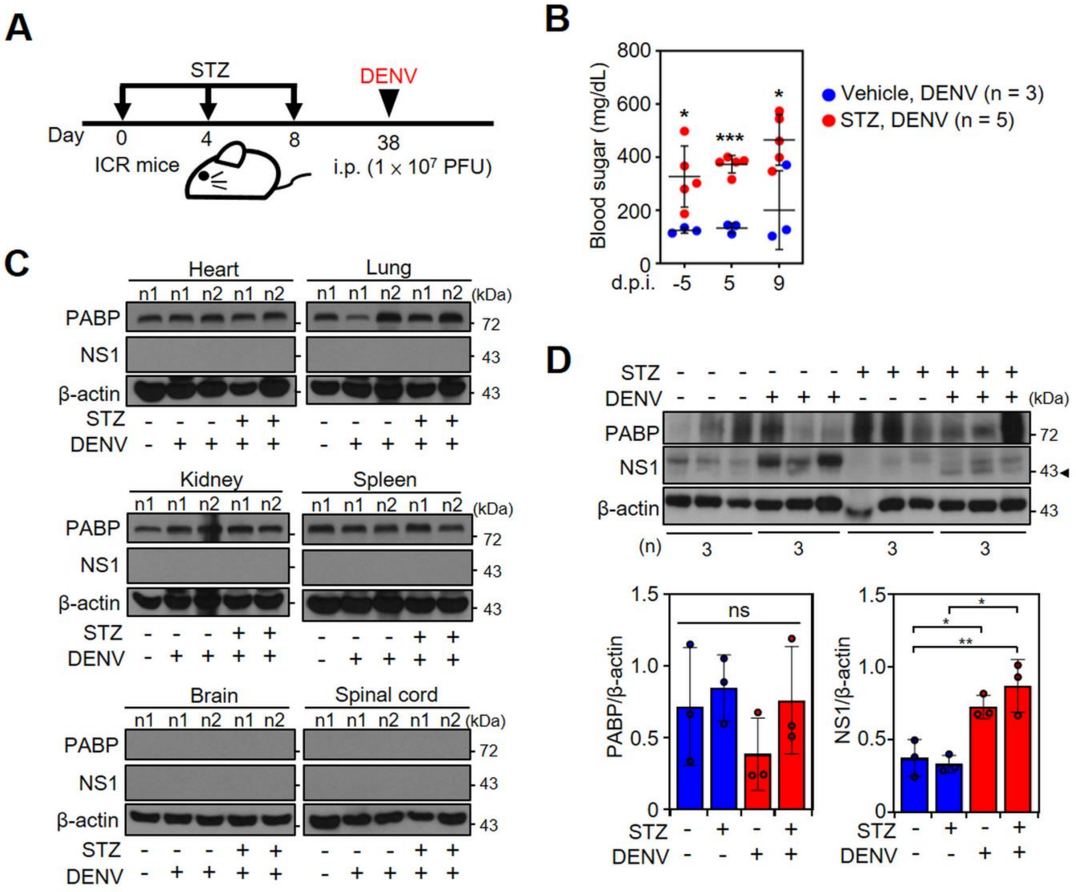

Supplemental Figure 6. DENV NS1 protein is detectable in adult mouse liver. (A) Adult ICR mice were i.p. injected three times with vehicle or STZ as indicated. At 30 days postinjection, the mice were i.p. inoculated with DENV 2. The blood sugar levels **(B)** of vehicle-treated and STZ-treated adult ICR mice were shown. Western blot showed the expression of PABP and viral protein NS1 of organs **(C)** and livers **(D)** of vehicle-treated and STZ-treated adult ICR mice with or without DENV 2 infection at 9 d.p.i. Quantitative data showed the mean ± SD of at least three mice. *$P < 0.05$, **$P < 0.01$, and ***$P < 0.001$. ns, not significant.

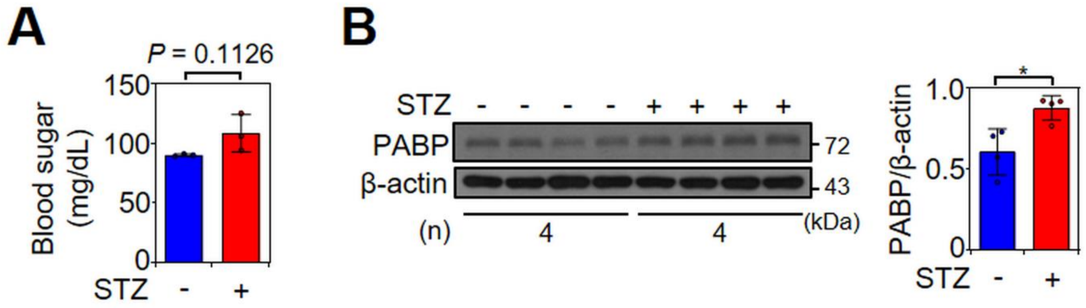

Supplementary Figure 7. Suckling mice from STZ-stimulated pregnant mice show increased blood sugar and liver PABP expression. The levels of blood sugar **(A)** and PABP expression **(B)** of the liver of 1-day-old suckling mice were examined. Quantitative data show the mean ± SD of at least three mice. * $P < 0.05$.

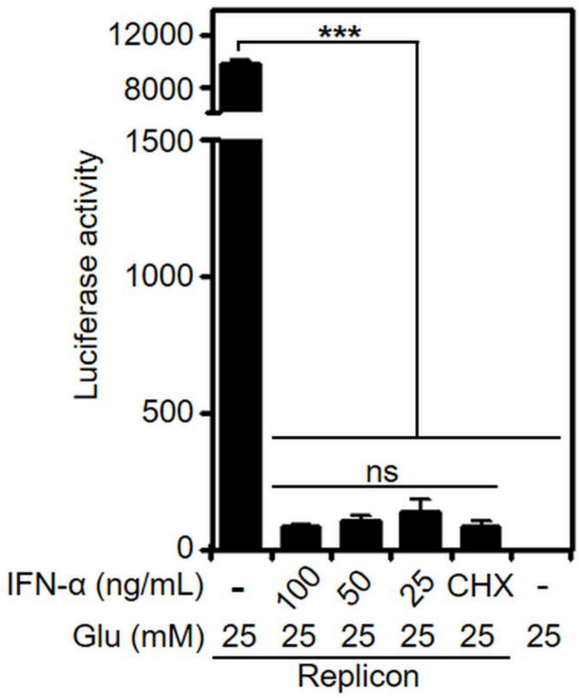

Supplementary Figure 8. Type 1 IFN reduces HG-enhanced translational activity. The luciferase activity assay showed translation activity in parental BHK-21 (line 6) and BHK-D2-Fluc-SGR-Neo-1 cells (replicons) treated with Glu 25 mM, with indicated concentrations of IFN-α or cycloheximide (CHX) for 48 hours. Quantitative data show the mean ± SD of three independent experiments. ****P* < 0.001. ns, not significant.

Supplementary Table 1. Characteristics of dengue group and a comparison group from 1998-2014 in Taiwan.

	Dengue group (Case) n = 30,944		Comparison group (Control) n = 123,776		
	n	(%)	n	(%)	*P* value
**Age, n (%)**					
Mean, SD	44.72,	19.06	44.72,	19.06	0.9912
median, iqr	46.51,	29.40	46.50,	29.39	
q1, q3	(30.09,	59.50)	(30.11,	59.50)	
min, max	(0.11,	98.17)	(0.01,	97.90)	
**Gender, n (%)**					1.0000
Male	15,510	(50.12)	62,040	(50.12)	
Female	15,434	(49.88)	61,736	(49.88)	
**CCI score, n (%)**					
mean, sd	0.68,	1.22	0.63,	1.22	<.0001
median, iqr	0,	1	0,	1	
q1, q3	(0,	1)	(0,	1)	
min, max	(0,	14)	(0,	16)	
**Comorbidity, n (%)**					
Hypertension	5,989	(19.35)	22,361	(18.07)	<.0001
Hyperlipidemia	4,331	(14.00)	14,864	(12.01)	<.0001
Diabetes mellitus	3,299	(10.66)	12,579	(10.16)	<.0001
Renal disease	817	(2.64)	2,943	(2.38)	0.0073
Peripheral vascular disease	498	(1.61)	1,875	(1.51)	0.2262
Cerebrovascular disease	1,499	(4.84)	5,816	(4.70)	0.2810
Myocardial infarction	193	(0.62)	719	(0.58)	0.3788
Congestive heart failure	592	(1.91)	2,428	(1.96)	0.5814

Note: The *P*-value of matched factor residence (including Kaohsiung, Tainan, and other cities in Taiwan) of these two groups is 1.0000 (not shown in the table).

Supplementary Table 2. The gene expressions of predicted transcriptional factors which target to the PABP.

Gene Symbol	Description	Glu 25 mM [b]Avg (log2)	Glu 5.5 mM Avg (log2)	[a]Fold Change
**AP-2α**	Transcription factor AP-2 alpha (activating enhancer binding protein 2 alpha)	4.84	4.43	1.33
**AP-2β**	Transcription factor AP-2 beta (activating enhancer binding protein 2 beta)	10.8	10.97	-1.13
**AP-2γ**	Transcription factor AP-2 gamma (activating enhancer binding protein 2 gamma)	9.33	9.28	1.03
**Brachyury**	T brachyury transcription factor	4.96	4.88	1.05
**E47**	Transcription factor 3	9.52	9.45	1.05
**Elk-1**	ELK1, member of ETS oncogene family	10.81	10.93	-1.08
**HFH-1**	HNF1 homeobox A	2.81	2.62	1.14
**Pax-5**	Paired box 5	5.88	5.77	1.08
**TBP**	TATA box binding protein	11.33	11.53	-1.14

[a]The fold-change is defined as the ratio of the difference between Glu 25 mM to the Glu 5.5 mM. The initial value calculated the values. [b]Avg, average.
